# Correction: Prognosis of patients with advanced bile tract carcinoma: assessment using the modified-Gustave Roussy Immune score (mGRIm-s) as a clinico-immunological tool

**DOI:** 10.1007/s00432-024-05829-9

**Published:** 2024-06-27

**Authors:** Yue Ma, Yuting Pan, Yue Li, Huafang Guan, Guanghai Dai

**Affiliations:** 1 Medical School of Chinese PLA, Beijing, 100853 China; 2 Department of Medical Oncology, The First Medical Centre, Chinese PLA General Hospital, Beijing, 100853 China; 3 Department of Medical Oncology, The Fifth Medical Centre, Chinese PLA General Hospital, Beijing, 100039 China; 4 Yingtan City People’s Hospital, Yingtan, 335000 China

Journal of Cancer Research and Clinical Oncology (2024) 150:247

10.1007/s00432-024-05771-w

In this article the co-first author tagging was missing for the author Yuting Pan. (Yue Ma, Yuting Pan have equally contribution to the article.)

The original article has been corrected.

